# Identifying and Mapping Levels of Active Engagement Within an Arts-Based Intervention: A Qualitative Analysis and Conceptual Development

**DOI:** 10.1007/s12529-026-10479-x

**Published:** 2026-07-27

**Authors:** Sarah A. Neller, Moroni Fernandez Cajavilca, Bob Wong, Julene Johnson, Lee Ellington, Jacqueline Eaton

**Affiliations:** 1 College of Nursing, University of Tennessee at Knoxville, Knoxville, TN, USA; 2 Rory Meyers College of Nursing, New York University, New York, NY, USA; 3 College of Nursing, University of Utah, Salt Lake City, UT, USA; 4 Division of Geriatrics, School of Medicine, University of California, San Francisco, CA, USA

**Keywords:** Alzheimer disease, Informal caregivers, Research methodology, Concept formation

## Abstract

**Background:**

Active engagement is crucial in psychoeducational interventions for care partners of persons living with dementia, yet measurement is limited. This manuscript explores participant engagement in an arts-based intervention designed to increase engagement in addressing dementia-related behavioral symptoms. The intervention uses multisensory activities, including caregiver-informed vignettes, to foster engagement and process caregiving experiences.

**Methods:**

Care partners of persons living with dementia (*n* = 9) participated in six focus groups.

Focus group data were analyzed using process coding to define and map patterns of active engagement across 27 intervention activities. The findings informed development of a conceptual model of active engagement.

**Results:**

Four levels of engagement were identified—*Participating*, *Clarifying*, *Contributing*, and *Applying*—and explored across participants, time points, and activities. The conceptual model illustrates that (1) antecedents contribute to (2) levels of engagement, leading to (3) the intervention’s hypothesized mechanisms of action, proximal outcomes (capacity to adapt, appraisal of caregiving demands), and distal outcomes (perceived stress, caregiver well-being).

**Conclusion:**

This manuscript provides a clear framework for operationalizing and measuring active engagement and demonstrates how engagement patterns can be conceptually linked to the intervention’s proposed mechanisms of action and outcomes. These findings provide transparency in reporting engagement patterns within an intervention, offering valuable insight into the components of active engagement and how these may be measured. The ability to track active engagement during intervention development and testing has the potential to improve our understanding of intervention dose and fidelity and how active engagement interacts with these to improve outcomes for participants.

## Introduction

Intervention development for persons living with dementia and their care partners has documented decades of progress across a wide variety of intervention approaches [1, 2]. The most effective psychoeducational interventions for care partners of persons living with dementia deliver education and skills by actively engaging care partners with the intervention [1–7]. The application of knowledge and skills are key components of active engagement, which is frequently accomplished through strategies such as role play, problem-solving, application of new knowledge, and discussion [1, 2, 6–9].

Given the dynamic, contextual, and complex nature of engagement, active engagement has yet to be consistently defined or systematically measured within behavioral interventions [10]. This limits understanding of the mechanisms by which active engagement interventions execute their effects and is a barrier to optimizing it within intervention delivery [1, 8, 9]. Therefore, researchers are tasked with considering the operationalization of active engagement and measurement for their studies, including the ways active engagement can be captured comprehensively (e.g., behaviorally, cognitively, and affectively) and to think through whether active engagement is a primary, secondary, or mediating variable [11].

A recent scoping review [12] identified six approaches used to encourage active participation in interventions for care partners of persons living with dementia including interventionist qualifications and participation, teaching methods, adapting to care partner needs, collaboration, peer sharing, and experiential learning. Of note, this review did not examine the outcomes of the approaches discussed, nor did it address methods for measuring and documenting active engagement [12]. Efforts have focused on tracking engagement objectively. For example, in asynchronous digital online behavioral interventions, participant time engaging with the intervention was monitored through usage and adherence data, such as online module completion [13]. Some interventions use subjective measures of engagement such as self-reported surveys or qualitative interviews to explore participants’ experience with the intervention or effort during the designed intervention activities [11, 14]. However, self-reported measures may not be an accurate reflection of active engagement because participants may over estimate effort [15]. It is often challenging to capture active participation during behavioral interventions using in-person or synchronous online formats because engagement is a dynamic process that is co-constructed among the participants and facilitator [10]. Objective measures alone do not accurately reflect the effort participants contribute during an intervention, such as sharing experiences, applying learned principles, and reflecting on future behavior change.

Arts-based approaches hold great potential to foster active engagement, as arts practice promotes imagination and creativity across a wide array of experiences [16, 17]. The arts have been incorporated into a variety of interventions to improve training, interpersonal and communication skills, problem-solving, preparation, and behavioral outcomes for individuals and communities across environments [18–20]. Specific arts activities within interventions include activating the senses, aesthetic engagement, cognitive stimulation, use of the imagination, and emotions [20]. Arts engagement occurs on a spectrum across a wide array of intervention approaches and settings, including everyday engagement for leisure, programs targeting health-related outcomes, and professionally led art therapies in clinical settings [16, 17]. A recent definition of arts participation includes two broad, inclusive components: first, the mode of participation (learning, consuming, participating, attending, etc.) and second, the art form (visual art, theatre, music, literary arts, dance, media) [17]. The term arts-based intervention describes the use of one or more art forms or art therapy in practice with the goal of achieving an improved outcome [20, 21]. Some note that the use of the arts for active engagement have failed to evaluate the process and level of active engagement [22, 23]. More recently, progress has been made on identifying mechanisms of arts engagement specific to health-related outcomes, which has promise to improve the methods for documenting mechanisms [16, 24]. The INgredients iN ArTs in hEalth (INNATE) framework provides broad guidance on potential mechanisms but lacks a systematic process to evaluate or explore levels of active engagement [24].

### Background of the Enhancing Active Caregiver Training (EnACT) Intervention

To enhance how care partners of persons living with dementia actively engage with training, the senior author [JE] developed the Enhancing Active Caregiver Training (EnACT) intervention. EnACT is an arts-based intervention aimed at helping dementia care partners better manage common behavioral symptoms associated with dementia [25, 26]. The goal of the EnACT intervention is to prepare for the realities of caregiving by exploring the lived experience of those caring for persons living with dementia. Because studies have shown that caregiving interventions are more effective when they combine practice with teaching, compared to focusing on didactic teaching alone [7], EnACT focuses on caregivers engaging with arts practices such as role play, improvisation, imagining future interactions, and sensory experiences to promote experiential learning, collaboration, and adapting. Unlike traditional didactic approaches that teach a “right” way to address behavioral symptoms of dementia, EnACT focuses on fostering interaction, collaboration, and mutual learning among participants.

EnACT’s foundation is rooted in arts-based principles that stem from Theatre of the Oppressed [27], ethnodrama [28–30], improvisation, and imagination [25]. It’s beginnings formed as community based ethnodrama research in which *Portrait of a Caregiver* was co-developed with caregivers of older adults and professionally produced and performed by trained theatre professionals [31]. After years of live performances, we sought to explore the use of *Portrait* to enhance advanced preparation for the challenges of caring for persons living with dementia [25, 26].

Development of this intervention was guided by the Imagined-Interactions Theory [32], and the Consequences of Dementia Caregivers’ Stress Process Model [33]. A more complete explanation of the theoretical framework can be found in [26]. The purpose of EnACT is to prepare in advance for the challenges of caregiving by practicing in safe spaces to reduce the gap between imagination and reality (decreased discrepancy), enhance the level of detail that one is aware of in behavioral encounters (increased multisensory detail), and promote preparation in advance of future care partner interactions (increased preparation) leading to increased ability to more accurately appraise and adapt to the demands of caregiving. These imagined interactions serve as the hypothesized mechanisms of action for the intervention and lead to the desired outcome of improving caregivers’ capacity to adapt, appraisal of caregiving demands, well-being, and perceived stress [26].

EnACT uses community-partner-informed vignettes from *Portrait* that portray common caregiving challenges related to behavioral symptoms of dementia [26]. Iterative review and revision of EnACT with multiple experts, including those with formal training and experience incorporating theatre across training settings, formalized vignettes and exercises that foster multisensory experiences to promote active methods to prepare for caregiving and the behavioral symptoms of dementia [25]. During the EnACT intervention, participants (a) **view** an evidence-based video portraying a caregiving scenario; (b) **practice** through small-group experiences that incorporate the five senses to enhance active engagement and support rehearsal of reactions, choices, and skills within a safe environment; and (c) **reflect** with the larger group on lessons learned, group choices, processes, and observations.

The EnACT intervention activities focus on experiential learning and include arts-based practices to imagine what may have happened before the vignette began, explore the perspectives of the care partners via character analysis, make improvised changes to the narrative, or participate in creative writing about a personal caregiving experience using a writing prompt. EnACT is an arts-based intervention because it uses specific arts practice to foster caregiver engagement with training and support groups. It incorporates arts-based practices from three art forms: performance (improvisation, role play, script reading), literary arts (fiction and storytelling), and visual techniques (film). During this phase of development, the intervention was delivered by the PI (JE), who has training in both theatre and teaching for adults [26]. Although standard measures of active engagement within behavioral interventions do not exist, understanding the role of active engagement within EnACT is critical to optimizing the intervention for caregivers. The purpose of this manuscript is to describe the specific methods used to:

1. Define active engagement within the EnACT intervention and identify levels of engagement,
2. Map how participants progressed through the active engagement levels over the course of the intervention, and
3. Develop a conceptual model to illustrate how active engagement contributes to the hypothesized mechanisms of action and expected behavioral outcomes of the EnACT intervention.

## Methods

During the development phase of EnACT, the study team conducted a series of focus groups among two groups of informal care partners of persons living with dementia. The study was approved by the University of Utah’s Institutional Review Board (#00135515). The methods and results of iteratively developing and refining EnACT are reported elsewhere [25, 26]. This manuscript reports on the concurrently conducted ad hoc analysis exploring active engagement within the intervention. Using the definition by Casey and Murray [34], we label this phase of our research as arts-based because the arts process used in EnACT was analyzed to understand how participants were engaging with the intervention and its meaning specific to active engagement. To analyze the data, we observed acts of creative expression through arts practices built into EnACT, and analyze the processes and products of these practices, including improvised narrative, role play, interaction with scripts, story creation, and the process of participants engaging in arts practice. This aligns with Casey and Murray’s [34] description of arts-based research as a method which emphasizes exploring lived social and cultural experiences through the use of arts processes. Participants used art practices to explore their lived experience of caring for persons living with dementia, and we analyzed how this process led to changes in levels of active engagement.

### Sample and Setting

Ten informal care partners of persons living with dementia were initially recruited through partnerships with the Alzheimer’s Association of Utah, the Utah Division of Aging and Adult Services, and the Time for Living and Care Study [35], which identified individuals interested in future research. Study details were shared via email, followed by outreach from a researcher (MFC, JE). Participants were offered $50 remuneration to attend three 90-min, virtual focus groups. Participants were assigned to one of two groups, depending on availability (half meeting in the afternoon and half meeting in the evening). The participants were divided into two groups (A and B).

### Data Collection and Analysis

The focus groups were led by the principal investigator (JE) and a trained researcher (SN) to test EnACT activities and gather participant feedback on their relevance, utility, and ease of participation. The PI (JE) and another researcher (MFC) used field notes to document observed participant behaviors and the researcher’s reflections to support a reflexive process. Focus groups were professionally transcribed and verified for accuracy by a researcher (MFC).

Transcripts were analyzed by an interdisciplinary team using a three-cycle process: (1) structural coding, (2) descriptive coding, and (3) pattern coding. Initial analysis aimed to identify what aspects of the EnACT intervention were effective and what needed improvement. Results from the initial analysis led to the ad hoc analysis, reported here, to explore levels of engagement.

#### Ad Hoc Analysis

During the initial process of analysis, the first author observed that the activities perceived as effective were those that contributed to a broader cycle of engagement. Effectiveness was often inferred from the volume and nature of participant responses—activities that generated only questions or complaints were perceived as less effective, whereas those that generated rich discussion were considered successful. This insight led recognizing the value of capturing and measuring participant engagement to better identify both effective components and areas requiring improvement. As a result, the study team decided to concurrently conduct this nurse-led, ad hoc analysis specifically focused on defining and mapping engagement within this intervention. Three focus groups, 2A, 2B, and 3A, were included in this analysis to focus solely on the groups that tested intervention activities.

##### Defining the Levels of Engagement

Coding first focused on participant verbal responses (descriptive and pattern codes) to each EnACT activity (structural code) [26]. The codebook was iteratively developed during analysis. As variability in both the quantity and quality of participant engagement emerged, levels of engagement were apparent, leading to the development of process codes. The codebook was continuously updated during team meetings to reflect this iterative process. Once the codebook was solidified, two nursing researchers (SN, MFC) coded the transcripts independently in NVivo 14 [36] and met regularly with the PI (JE) to resolve any discrepancies.

##### Mapping the Patterns of Engagement

After coding levels of engagement, the patterns were mapped to visualize engagement within each focus groups. As all focus groups varied in number of coded segments, each group was standardized by creating a percentage of coded segments (i.e., participant phrases assigned engagement codes). Tableau software [37] was used to develop time series charts. Additionally, participant engagement patterns were analyzed over time within the focus group by assigning the levels of engagement to the coded segments by activity prompt and tracking participants’ progression through each activity. This allowed us to visualize engagement dynamics across discussion group size (both small and large groups), vignette type, and EnACT activity. Engagement patterns were further triangulated from three activities that were tested in all three focus groups: (a) “Other Perspectives” in which participants considered what the caregiver, portrayed in the vignette, was thinking or feeling; (b) “Changing Stories” in which participants considered what needed to happen for the depicted situation to change; and (c) “Making Choices” in which participants made different choices based on other perspectives. Finally, the patterns of engagement were integrated with the hypothesized mechanisms of action within the theoretical framework to create the EnACT Conceptual Model of Active Engagement.

## Results

Nine care partners of persons living with dementia participated in the study with four in focus group A and five in focus group B (*n* = 5) with each group completing three focus groups held virtually over Zoom. Although ten participants enrolled, one withdrew due to a change in family circumstances prior to the first session. Participants had a mean age of 61 years old and were primarily female (89%), caring for a spouse/partner (67%), and had been providing care for 3 to 5 years (56%) or longer (33%). A total of 27 activities across focus groups 2A, 2B, and 3A were analyzed using a combination of between one to seven activities for each of the five chosen vignettes. Using the developed codebook, a definition of active engagement within EnACT was created: Active engagement occurs when participants apply the knowledge learned in caregiver training and support groups to the act of making decisions, verbalizing, questioning, or cognitive processing.

### Defining the Levels of Engagement

The four ordinal levels of engagement—*Participating*, *Clarifying*, *Contributing*, and *Applying—*were developed to convey the depth of participant engagement, without the implication that there is one right or wrong way of participation. Examples of the levels of engagement using descriptions, exemplar quotes, and percentages are provided in Table 1.

1. *Participating*: participants offered generalized verbal responses, agreed or disagreed with other participants, or commented about the caregiving vignette.
2. *Clarifying*: participants sought further instruction about the activities by asking clarifying questions or expressing confusion about the prompts.
3. *Contributing*: participants responded to activity prompts within the context of the care partners portrayed in the vignette. This means they answered the question prompts based on their observations in the vignette.
4. *Applying*: participants responded to activity prompts by sharing their personal caregiving experiences extending beyond the context of the care partners portrayed in the vignette. Participants frequently related to the caregiver’s thoughts and feelings, responding based on how they themselves would feel in similar situations, and used this connection to apply the caregiving scenario to real-world experiences.

### Mapping the Patterns of Engagement

**Fig. 1:**
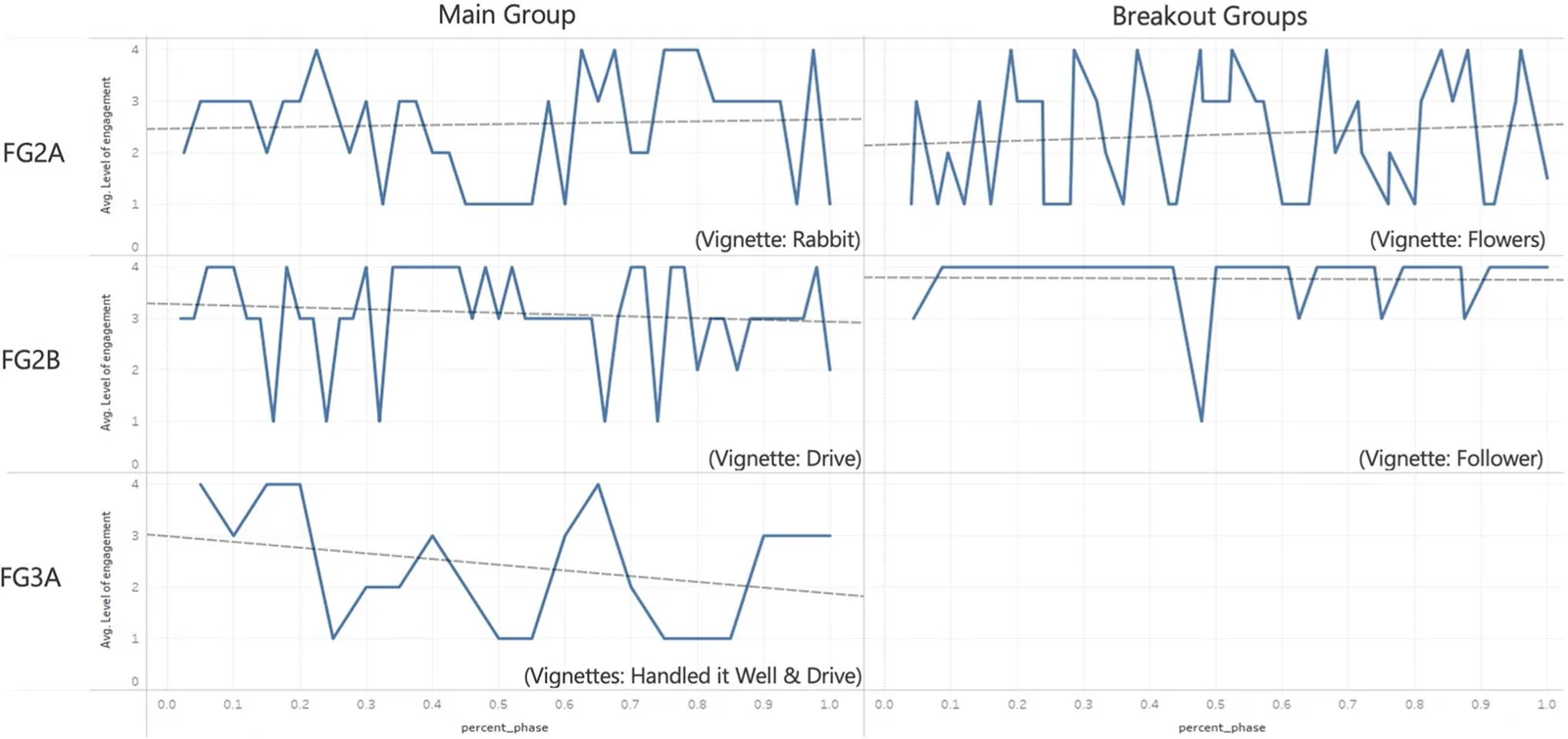
Levels of engagement over time. Note. The column on the left, Main Group, represents when all participants met together. The column on the right represents when the focus groups were split into two smaller groups and discussed the same vignette. The *X*-axis shows the focus group time broken into 10% increments. On the *Y*-axis, the 1–4 numbers correspond to the engagement codes from the codebook—*Participating*, *Clarifying*, *Contributing*, and *Applying*. The blue line represents the coded segments over time, and the dotted trend line indicates the average level of engagement over time

**Fig. 2:**
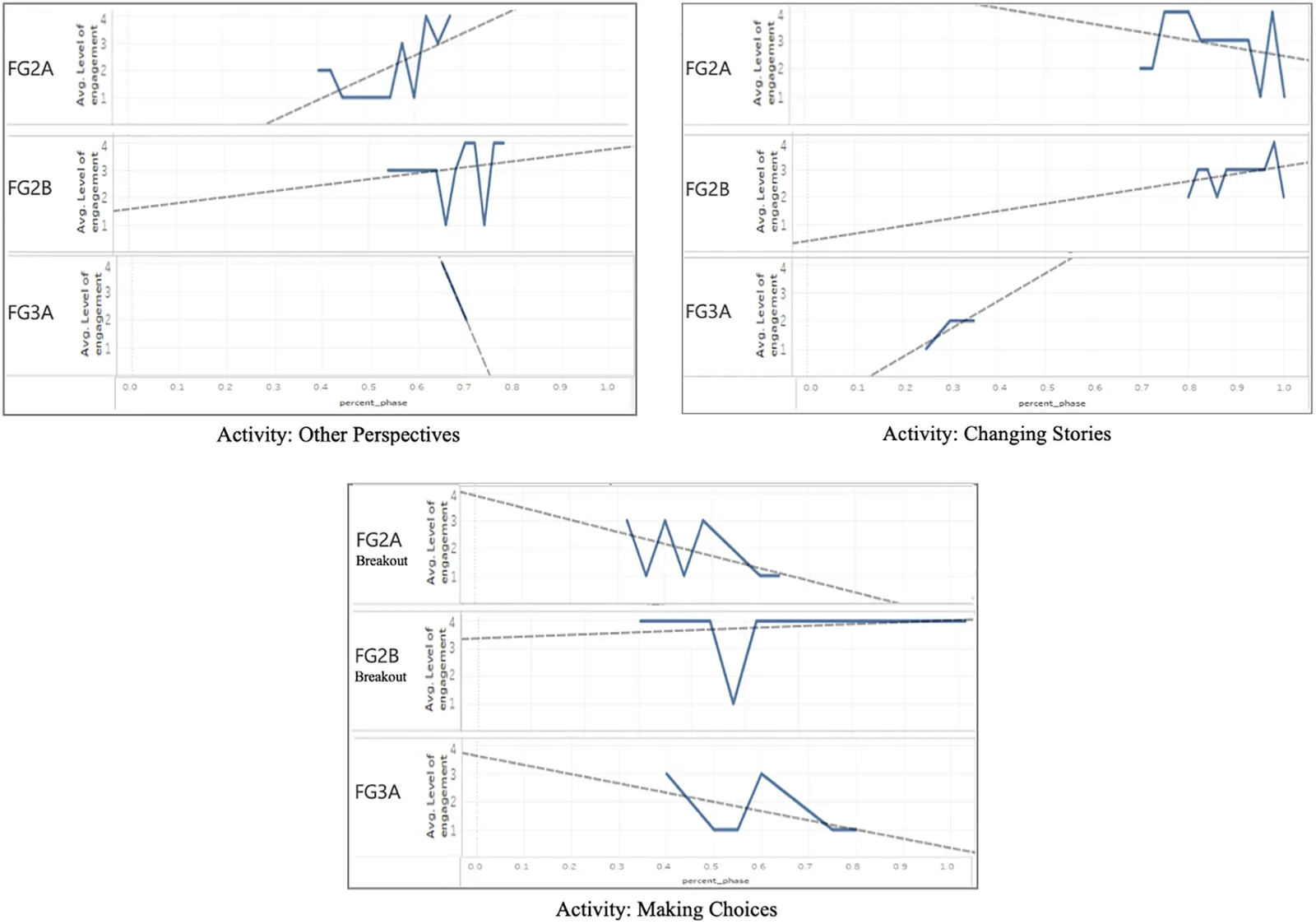
Levels of engagement by activity

The analysis of the time series plots for each focus group helped identify the patterns of engagement over time (Fig. 1), and highlighted the activities within each group that sparked the most active engagement (Fig. 2). Engagement levels varied based on vignette content, activity type, and the timing of activities within the session.

All participants were involved at nearly all levels of engagement during the focus groups. While the overall engagement (e.g., number of coded segments) was similar between focus groups 2A (86 segments) and 2B (81 segments), the percentage of engagement levels varied by *Participating* (30% vs. 7%), *Clarifying* (13% vs. 4%), *Contributing* (38% vs. 35%), and *Applying* (19% vs. 54%). This indicates that focus group 2A had more overall engagement and focus group 2B had deeper levels of engagement (e.g., higher percentage of *Applying*).

#### Mapping Engagement Over Time

Patterns of engagement over time are presented in Fig. 1. When comparing the overall engagement of focus groups 2A and 2B when the activities were offered with all participants together in the main Zoom room, participants in both groups started by asking *Clarifying* questions or *Contributing* to the discussion by answering the activity prompts related to the vignette they viewed. Participants were moved to the breakout rooms to explore a new vignette and a different set of activities. Focus group 2A started off similarly by asking questions, contributing, and validating each other and then moved through all four levels of engagement. In contrast, when focus group 2B moved into breakout rooms, participants moved through only two levels of engagement (*Contributing* and *Applying*). They moved back and forth between the caregiver’s experience portrayed in the vignette and their own lives, exhibiting deep levels of engagement within the breakout rooms. During focus group 3A, some of the activities used during focus group 2A were revisited to see if using a different vignette (e.g., “Drive”) that resonated well with the other participants in focus group 2B might promote deeper levels of engagement. While both focus groups exhibited all levels of engagement, focus group 3A had very little *Applying* during the Drive vignette (6%), in contrast to focus group 2B, which had much higher levels of *Applying* (36%) during the same vignette.

#### Mapping Engagement by Activity

The research team encountered challenges coding engagement within the activities and assessing if the activities were effective—whether participants understood and followed the prompts and contributed to discussions about the caregiver in the vignette. As caregivers began applying the content to their own experiences, discussions often extended beyond the structured activity, with participants sharing personal narratives. This fostered peer-to-peer dialogue driven by shared experiences, naturally moving the conversation beyond the activity prompts. The interactions frequently shifted away from the specific behavioral topic in the vignette, as caregivers addressed other challenges or exchanged practical advice. These coded segments were attributed to the activity that originally initiated the discussion, as it appeared to be a meaningful catalyst for engagement among caregivers.

Mapping the flow of engagement within these activities allowed us to track participants’ progression through the identified levels of engagement over time. Figure 2 depicts the results of triangulating three activities that were tested across focus groups 2A, 2B, and 3A. The activity on the left of the figure, “Other Perspectives,” resonated with focus group 2A and focus group 2B. Participants demonstrated all levels of engagement here. Focus group 2A began by *Clarifying* but then gained momentum and began *Contributing* and *Applying* near the end of the activity. Focus group 2B began by *Contributing* and ended with participants *Applying*. However, the “Other Perspectives” activity was not clear for participants in focus group 3A, and it did not facilitate engagement. The activity “Changing Stories,” facilitated high levels of engagement for focus groups 2A and 2B. In focus group 2A, participants began by *Clarifying* the instructions and then entered a period of *Applying* and *Contributing*. In focus group 2B, participants also began by *Clarifying*, then moved into *Contributing* and *Applying*. However, this activity did not move beyond *Participating* and *Clarifying* in focus group 3A. In the activity “Making Choices” (see Fig. 2), focus group 2A started by *Contributing*, but there were many questions and points of confusion, and the activity never gained momentum. In contrast, the same activity seemed to immediately resonate with focus group 2B as demonstrated by nearly all *Applying* during the entirety of the activity. In focus group 3A, participants began by *Contributing*, but they again experienced confusion and needed clarification with this activity.

### The EnACT Conceptual Model of Active Engagement

**Fig. 3:**
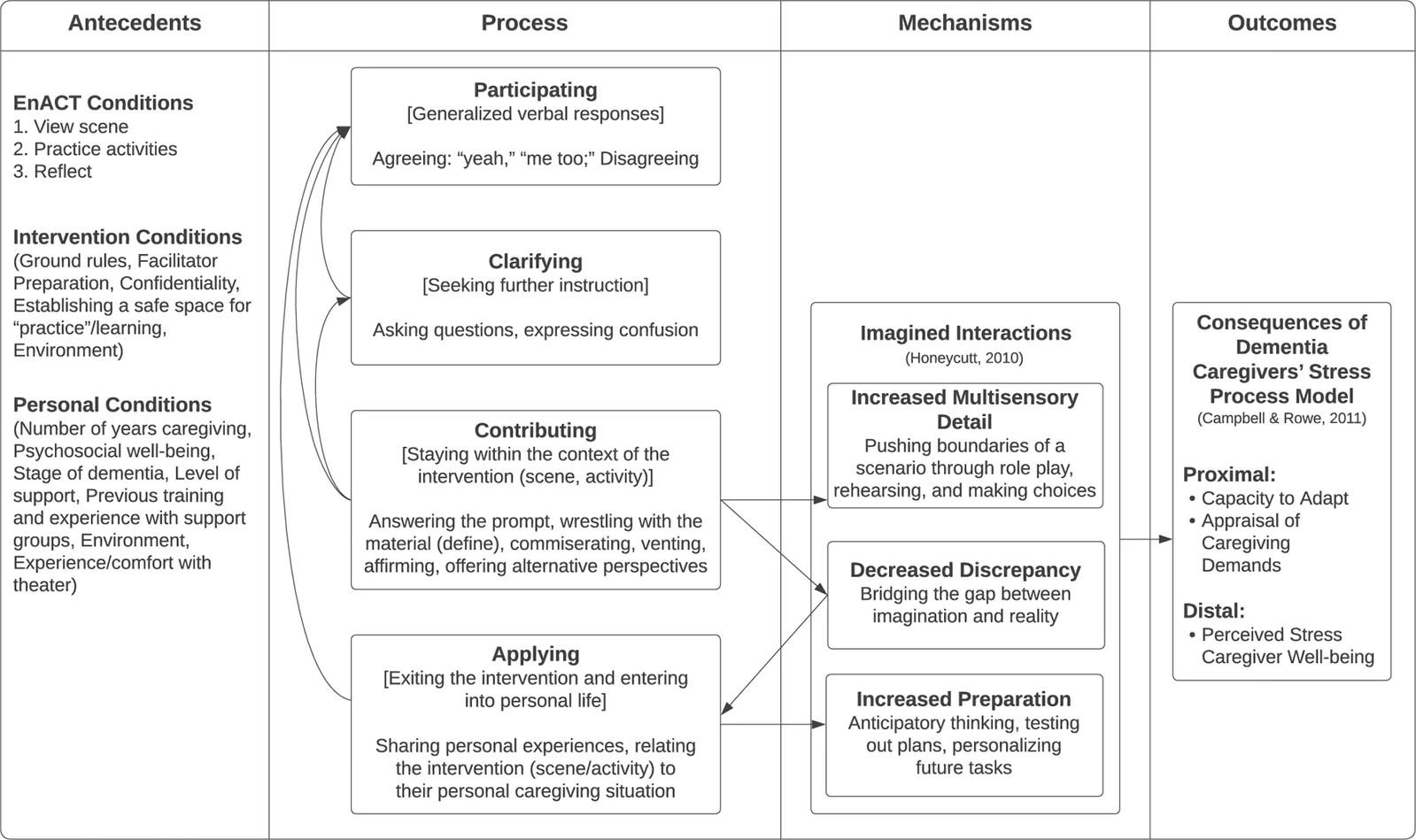
EnACT conceptual model of active engagement

To address the second aim, the theoretical framework of EnACT (see [26]) was expanded to develop a conceptual model to position the levels of engagement with the hypothesized mechanisms of action and outcomes of the EnACT intervention (Fig. 3).

The conceptual model starts with the antecedents of the intervention, which help contextualize each participant’s ability to engage. These antecedents include the components of the intervention (e.g., view, practice, reflect), engagement conditions, and personal conditions. Engagement conditions include facilitator preparation, creating a safe space for interaction, and the environment (e.g., in-person or online platforms like Zoom). Personal conditions encompass factors such as caregiving experience, psychosocial well-being, dementia stage, prior training, and the participant’s personal environment.

The second column of the model outlines the process of engagement through four levels: *Participating*, *Clarifying*, *Contributing*, and *Applying*. The first two levels, *Participating* and *Clarifying*, are foundational for the deeper levels of *Contributing* and *Applying*. These initial stages help establish group belonging and ensure participants feel validated, reducing confusion and preparing them for more active involvement by *Contributing* and *Applying*.

These levels of engagement are linked to three hypothesized mechanisms of action (column 3), derived from Imagined-Interactions Theory [32]. The research team hypothesizes that the process of *Contributing*, through experiential learning, increases multisensory detail and reduces discrepancies between imagined caregiving scenarios and reality. This bridging of the imagination-reality gap allows participants to better prepare for caregiving challenges. As participants move to the *Applying* level, they engage in anticipatory thinking, test plans, and personalize their experiences, further reducing discrepancy by connecting the scenarios to their own lives. This process has the potential to reduce perceived stress and enhance the caregivers’ ability to assess and adapt to caregiving demands, promoting better well-being [33].

## Discussion

Active engagement is a central component and outcome of the EnACT intervention. This analysis (a) defined active engagement within the intervention, (b) developed the levels of active engagement during intervention sessions, and (c) created a model conceptualizing how the levels of active engagement contribute to intervention outcomes for care partners of persons living with dementia. During the intervention sessions, participants moved across all levels of engagement, which demonstrates growth as participants were willing to ask questions, interact with each other, wrestle with intervention activities, and then share their own caregiving journey. This dynamic engagement highlighted the value of using the activities as a platform to facilitate rich, participant-driven discussion.

Existing literature underscores that care partners desire understanding and recognition in their role [38]. For care partners of persons living with dementia, interacting with others in group-based settings can enhance well-being and provide social and emotional support by offering a sense of togetherness [39–41]. Findings from the current analysis suggest that vignettes used in partnership with discussion prompts may serve as effective catalysts for engagement. By presenting hypothetical caregiving scenarios, these prompts encourage participants to reflect on and relate the content to their own caregiving experiences. This process fosters personal application and reciprocal sharing, which collectively deepens engagement and facilitates the development of meaningful peer connections within the intervention setting. Such interpersonal interactions, stimulated through structured engagement activities, may also contribute to an expanded and more diverse social network for caregivers—an important factor linked to enhanced well-being [42].

### Challenges in Defining and Analyzing Engagement

Previous research has highlighted that identifying the key strategies and the underlying mechanisms to meaningfully engage care partners is likely to enhance caregiver outcomes [2, 8, 43]. Although there is a growing body of literature focused on measuring engagement in mobile and digital health interventions [44, 45], guidance remains limited regarding how to assess engagement in in-person interventions. Bijkerk et al. [11] offer valuable recommendations for measuring engagement in behavioral interventions; however, many of these approaches rely on self-reported questionnaires, usage metrics, or post-session qualitative interviews rather than real-time, objective observation. While self-report tools can provide insights into participants’ perceptions of adherence and effort [11, 15], they fall short in capturing active interaction with the intervention or charting engagement trajectories across time—an important precursor to outcome assessment. Moreover, self-report data can be unreliable, as participants frequently overestimate their involvement or time spent engaging in the intervention [15]. A small number of studies have explored engagement patterns using self-report and app-based interaction data [45, 46], yet robust frameworks for capturing active engagement in in-person formats remain underdeveloped. Assessing active engagement without relying solely on self-report presents a unique challenge, particularly in group-based, in-person settings. There is a pressing need for tailored tools and approaches to guide facilitators and researchers in measuring dynamic patterns of engagement within live intervention contexts.

#### Defining Levels of Engagement

During analysis, it was challenging to define levels of engagement, systematically code participant responses during activities, and interpret engagement data. Participants’ engagement with structured activities often evolved into peer-led discussions, blurring the line between guided prompts and organic conversation. This shift illustrates the challenge of defining active engagement, as structured prompts frequently serve as catalysts for broader, participant-driven dialogue. These complexities highlight the need for more precise conceptual and methodological approaches to assessing active engagement in behavioral interventions.

#### Mapping Patterns of Engagement

During the engagement mapping process, the research team was challenged in determining how to interpret whether a vignette or activity resonated with the participants and should be retained for future iterations of EnACT. While the trend lines in Figs. 2 and 3 provide a high-level view of engagement across a session or activity, they should be interpreted with caution. They represent overall group-level engagement and do not account for individual participation or progression through different levels of engagement over time. Additionally, the trajectory of the trend line does not always indicate the depth of overall engagement. For example, in focus group 3A’s “Changing Stories” activity, the trend line exhibits a steep positive slope; however, the activity did not facilitate contribution or application. Although the group progressed from *Participating* to *Clarifying*, suggesting an upward trajectory of engagement, the discussion remained at the level of *Clarifying* rather than advancing to *Contributing* or *Applying* the material. While *Participating* and *Clarifying* are integral components of EnACT, we hypothesize that *Applying* is a crucial mechanism for activating imagined interactions, ultimately leading to positive caregiving outcomes.

Engagement levels may also have been impacted by the iterative revision of the intervention that occurred between each focus group, leading to clearer intervention instructions and enhanced engagement, while an improvement in activity engagement was frequently seen from focus group 2A to focus group 2B (see Fig. 2: “Changing Stories” and “Making Choices”), there was not always an improvement in the engagement from focus group 2B to focus group 3A (see Fig. 2: “Making Choices”). For example, though focus group 2B was presented with the Drive vignette and activities as part of their second meeting together and focus group 3A was presented with the Drive vignette and activities in their third meeting, focus group 3A had a very low percentage of *Applying*, suggesting that longer time spent with group members does not always lead to deeper levels of application. This finding may be because the selected vignettes resonated differently between the focus groups, group dynamics varied due to participant differences, or that the activity instructions still needed refining.

### Using the Conceptual Framework to Explore and Operationalize Active Engagement in EnACT

Understanding active engagement necessitates consideration of both internal and external factors that influence caregiver participation. Current literature emphasizes the importance of identifying predictors and facilitators of active engagement to enhance intervention effectiveness [11, 12]. Within the study, the antecedents component of the conceptual framework accounted for these influences, shaping how participants engaged with the intervention. Consistent with the findings of Liu and Gellatly [47], progression through the levels of engagement required participants to be willing to interact and share personal experiences. Moreover, the degree of engagement among older adult participants may have been influenced by their prior exposure to caregiver training and support groups. Given that EnACT is an arts-based intervention, a lack of familiarity with creative or expressive modalities may have also altered or limited participants’ engagement [47]. These findings underscore the importance of tailoring interventions to accommodate diverse experiential backgrounds and learning preferences [12].

While EnACT fosters connection and shared understanding like traditional support groups, it extends beyond peer support by presenting external caregiving scenarios, encouraging alternative perspectives, and prompting personal reflection [40, 48]. Rather than prescribing specific educational strategies, EnACT focuses on developing transferable skills aligned with the hypothesized mechanisms of imagined interactions. This distinction is critical: instead of learning predefined solutions, caregivers cultivate cognitive and emotional regulation skills that can be applied to future caregiving challenges. By prioritizing skill development over directive instruction, EnACT equips caregivers to navigate dementia caregiving with greater adaptability. While EnACT’s problem-solving approach enhances active engagement for many caregivers, it may not align with all learning styles. Barriers to participation include discomfort with sharing personal experiences, environmental challenges (e.g., managing care responsibilities while attending), and varying learning preferences. Some caregivers prefer structured instruction over an interactive approach, while others find imagining future scenarios overwhelming. Since participants did not know each other beforehand, rapport-building likely influenced comfort levels.

The antecedents of engagement should be viewed as modifiable, as factors that facilitate active engagement for one participant may serve as barriers for another. The conceptual model provides a framework to analyze these factors, allowing for a deeper understanding of how engagement evolves. Future iterations may benefit from integrating EnACT into existing caregiver groups, where pre-established relationships may encourage more open engagement in discussions.

### Limitations

Several limitations related to the measurement and interpretation of participant engagement must be considered. Only verbal engagement was analyzed without body gestures and facial expressions from the Zoom recordings. During this preliminary phase, the focus was on testing as many activities as possible; therefore, there is not a direct comparison of activities for each vignette per focus group. Given the natural flow of the sessions, activities were not rigidly carried through to completion, leaving open the possibility that with additional time, participants may have elicited further discussion. However, in focus group A, the research team often had to move to the next activity due to limited dialogue; whereas in focus group B, discussion occurred more naturally, which facilitated transitions to additional activities. Observations of group dynamics demonstrated that the different make-up of participants in each group influenced interaction and activity response.

While some activities involved participants reading a scripted vignette before engaging with the activity prompts, this component was not considered a form of engagement and was not included in the coding framework. This decision aligns with the conceptualization of active engagement, which prioritizes participants’ active reactions and interactions with the material rather than more passive forms of participation. Although reading a script does indicate willingness to engage and involvement in the session, future studies should more fully explore including this as a component of the levels of engagement. It remains unclear whether participants must progress through all identified levels of engagement during the EnACT intervention to experience positive outcomes, or how varying levels of engagement influence those outcomes. As no self-reported engagement measures were used, participants’ internal cognitive or emotional processing of the intervention content was not assessed, potentially resulting in an incomplete picture of overall engagement. It is possible that some participants engaged meaningfully on a reflective level without expressing this outwardly during group sessions. Furthermore, how participants applied or integrated the skills learned during the intervention into their caregiving practices outside of the structured sessions was not assessed. Future research can incorporate both self-report and follow-up measures to better understand internal engagement and real-world application of intervention content.

### Next Steps and Implications

The engagement levels can be used by facilitators of EnACT moving forward to gauge active engagement within their groups, which will help increase rigor and reproducibility in future testing. By providing a structured way to assess engagement, facilitators can better understand participant involvement and make necessary adjustments to improve the intervention’s effectiveness. This structure will also promote analyzing individual engagement trajectories within EnACT to better understand how participants interact with and apply the intervention content. Moving forward, this conceptual model will be used to compare the engagement observed within these focus groups to participants’ level of engagement over time in the current EnACT randomized controlled trial. Additionally, further measures of engagement will be incorporated by assessing whether a participant is using the intervention knowledge or skills as intended between session exercises and surveys.

This work contributes to the science by (1) increasing transparency in how to define and map patterns of active engagement within an intervention and (2) demonstrating how the patterns of engagement can be conceptually linked to the intervention’s proposed mechanisms of action and outcomes. By establishing these connections, the research provides a framework for understanding how active engagement influences the success of the intervention, offering valuable insights for future research and practice. This analysis addresses current recommendations to define success within an intervention by more than usage data and explore patterns of optimal engagement within behavioral interventions [12, 49]. The four levels of active engagement patterns demonstrate different forms of participation that span a range of approaches to interacting with the intervention activities. They demonstrate the ways that attendees respond to facilitators and content, and document levels of depth, understanding, application, and connection. Better understanding the different forms of active engagement will make it possible to operationalize and more accurately measure the concept as the EnACT intervention moves to address implementation in naturally occurring settings, such as existing support groups. The steps outlined in this manuscript can be adapted by other researchers to evaluate participant engagement within their interventions. This adaptability ensures that the methodology can be applied broadly across various contexts and studies. This work can contribute to improved caregiver outcomes by helping other researchers challenge their analysis of participant engagement, so that describing participant engagement becomes a standard in intervention testing and reporting.

## Conclusion

This manuscript details the specific steps taken to analyze and track participant engagement patterns during the development phase of the EnACT intervention, providing insights into how engagement evolved throughout the intervention. This study offers a framework for defining and measuring active engagement within an arts-based intervention and demonstrates how engagement patterns align with the intervention’s proposed mechanisms of action and outcomes through a conceptual model. These findings increase transparency and provide a foundation for future research on optimizing engagement in behavioral interventions.

## Figures and Tables

**Table 1:** Engagement level descriptions and outcomes by focus group

Engagement level	Description	Exemplar quote	Percentage of engagement per group		
			2A	2B	3A
1. *Participating*	Offering generalized verbal responses: agreeing or disagreeing with other participants comments related to the vignette	“Yeah, I’d agree because I had a hard time reading it. It seemed like I was-- I didn’t know what I was saying, and then it jumped to another thing.” (Emily, 2A) That is a very good point, that’s a very realism. (Lena, 2B)	34.4%	18.2%	33.3%
2. *Clarifying*	Asking clarifying questions or seeking further instruction about the activities or prompts	“Yeah, I’m trying-- with John I’m trying to figure out what it is that you are trying to point out here.” (Irene, 2A) “What are you looking for again? I’m sorry because I’m like brain-dead. But what is it you’re asking exactly?” (Lena, 2B)	14.0%	4.5%	19.0%
3. *Contributing*	Answering the activity prompt; response relates directly to the vignette	“So in my mind if she got her driver’s license after the fifth time, she has a driver’s license. It’s obvious that she shouldn’t have a driver’s license. So Hannah’s going to have to make moves to get that driver’s license like taken away somehow.” (Mabel, 2B) “I would say the person that lives with her or is her caregiver realizes the problems that are going on. Whereas the sister and brother, probably don’t spend nearly as much time, don’t see the problem and think everything’s okay.” (Eva, 2B)	33.3%	29.5%	28.6%
4. *Applying*	Transitioning from the activities to sharing personal caregiving experiences	“In my personal experience, [my wife] would have started looking for the rabbit, and then got completely derailed because I think her attention span wouldn’t be long enough to look for a rabbit for more than 5 or 10 seconds. And then something else would take off.” (John, 2A) “Well, I think that also in that first sentence I thought, you know, that’s a typical caregiver in my understanding is that it’s so fragmented. It’s like, oh I want to tell you this I want to tell you that, but I need to hurry through this. I want to get to a point. And that’s what I thought when I first-- we first came across that I thought that’s the way I sound sometimes.” (Florence, 2B)	18.3%	47.7%	19.0%

## Data Availability

The data that support the findings of this study are not publicly available due to small sample size.
